# Field validation of an eDNA assay for nutria illuminates a role in invasive species management

**DOI:** 10.1002/ece3.11416

**Published:** 2024-05-23

**Authors:** Stephanie S. Coster

**Affiliations:** ^1^ Biology Department Randolph‐Macon College Ashland Virginia USA

**Keywords:** coypu, environmental DNA, eradication, monitoring, *Myocastor coypus*, nutria

## Abstract

Nutria, or coypu (*Myocastor coypus*), are invasive semi‐aquatic rodents present across the United States, Europe, and Asia. Despite successful eradication efforts in certain areas, nutria have resurged in the mid‐Atlantic USA, underscoring the need for advanced monitoring tools. Environmental DNA (eDNA) has emerged as a promising technique for species detection and monitoring. Here, an eDNA assay for nutria using qPCR was field‐validated in Virginia, USA, showcasing its potential as a tool for post‐eradication monitoring. The findings reveal an association between water levels and detection of nutria eDNA, highlighting the importance of water levels in nutria behavior. A painted turtle assay was introduced to confirm nutria absence and demonstrate the potential of passive sampling. The study showcases the sensitivity and efficiency of eDNA assays, emphasizing their value for monitoring and verifying invasive species eradication.

## Graphical abstract

An eDNA assay for nutria was field‐validated in Virginia, USA, showcasing its potential as a tool for post‐eradication monitoring. The findings reveal an association between water levels and nutria eDNA detection, highlighting the importance of water levels in nutria behavior. The study showcases the sensitivity and efficiency of eDNA assays, emphasizing their value for monitoring nutria and verifying eradication.

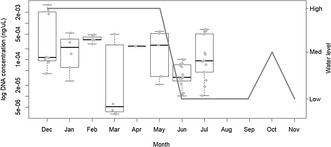

## 1 INTRODUCTION

Nutria, also known as coypu (*Myocastor coypus*), are large semi‐aquatic rodents that are invasive across the United States, Europe, and Asia (Carter & Leonard, 2002). Initially introduced from South America to the United States for fur farming during the late 1800s, nutria established wild populations throughout the country (Carter & Leonard, 2002). Notable hotspots of nutria invasion include areas near the Gulf of Mexico, the Pacific Northwest, and the mid‐Atlantic region of the USA (Mangan et al., 2023). Their foraging habits involve digging through soil in search of roots and rhizomes and result in the destruction of wetland vegetation (Jojola et al., 2005; LeBlanc, 1994). This, in turn, endangers a valuable natural resource crucial for flood protection, food production, recreation, and habitat preservation.

The reproductive potential of nutria is high, with one model predicting that a single breeding pair can result in up to 16,000 individuals within just 3 years (Sheffels & Sytsma, 2007). Consequently, the detrimental impact of nutria on the ecosystem can be substantial. Recognizing the adverse effects of nutria within the mid‐Atlantic region, the U.S. Fish and Wildlife Service invested $20 million to eradicate nutria from the Chesapeake Bay. After two decades of intensive management, Maryland declared them eradicated in 2021 (Anderson et al., 2022; Pepper et al., 2017). However, less than a year later, nutria are once again advancing north towards the Chesapeake Bay, migrating from North Carolina through Virginia (Kimberlin, 2021; Messina, 2021; Williams, 2021).

The elusive nature of nutria presents a significant challenge in management and eradication efforts. These destructive foragers are primarily active from dusk through dawn and reside in concealed burrows, making detection challenging (Jojola et al., 2005; LeBlanc, 1994). Traditional monitoring approaches involve on‐the‐ground scat and sign surveys, or the installation of wooden platforms in the water or on land, coupled with camera traps or hair snares (Baker & Clarke, 1988; Pepper et al., 2017). Collection and analysis of fecal matter and hair from these platforms aid in distinguishing nutria from native species like muskrat or otters (Pepper et al., 2017). However, constructing and maintaining these platforms demands a substantial investment of time and effort.

Early detection and eradication are paramount in halting the spread of nutria, as the costs associated with control and containment escalate dramatically as population numbers surge (Holden et al., 2016). There exists a brief window where the population size is still manageable enough to make eradication a viable option if individuals can be detected (Drake, 2005). In Virginia, a resident population has persisted near Norfolk for decades, where numbers are controlled by sub‐optimal habitat. Yet, nutria are now dispersing, with the first individuals tracked north of the James River in 2020. This region provides prime habitat and poses a threat of repopulating the Chesapeake Bay. Land managers in this area are acutely aware of the problem, and a swift, united effort could still enable containment. At this stage of invasion, managers are eager for innovative tools to track nutria.

Over the past decade, environmental DNA (eDNA) has emerged as a powerful and promising tool for assessing species presence (Ficetola et al., 2008; Goldberg et al., 2011), estimating biomass/abundance (Klymus et al., 2015; Takahara et al., 2012), and identifying community composition of freshwater species (Elbrecht & Leese, 2017). eDNA methods involve capturing and amplifying trace DNA found in the environment, which is shed through mucous, excrement, tissue, or decay (Turner et al., 2015). This non‐invasive technique allows the detection of species without the need for direct observation or physical sampling. eDNA can be extracted from various sources, such as soil or sediment (Turner et al., 2015), water (Goldberg et al., 2011), swabs (Klocke et al., 2017), and feces (Zeale et al., 2011). Quantitative PCR (qPCR) is the primary method used to amplify eDNA products in species‐specific assays, while high‐throughput sequencing and metagenomics are employed to understand community composition (Thomsen & Willerslev, 2015).

An eDNA qPCR assay for detecting nutria was initially developed in Japan (Akamatsu et al., 2018) and recently lab‐validated using nutria tissue samples from various states in the United States, including California, Louisiana, Oregon, Maryland, and Virginia (Mangan et al., 2023). The assay was tested against co‐occurring species to confirm specificity, and subsequent field validation took place in Oregon (Mangan et al., 2023). Current recommendations emphasize re‐validating eDNA assays in new geographic areas that might encompass genetic variation for quality control (Langlois et al., 2021; Thalinger et al., 2021). Additionally, understanding how detection varies over time and space is valuable for optimizing monitoring strategies and enhancing the accuracy of nutria presence assessments. Therefore, the objectives of this study were to: (a) field‐verify the eDNA assay in several ponds with putative nutria presence and absence in Virginia, USA, and (b) to explore variations in eDNA detection by month or time of day in a selected focal pond.

## 2 METHODS

### 2.1 Assay testing

Two tissue samples were obtained from nutria euthanized in Virginia from the United States Department of Agriculture (USDA) Animal and Plant Health Inspection Service (APHIS). As tissue samples were collected from nutria killed via legally authorized control methods, sample collection was exempted from Institutional Animal Care and Use Committee review. It was unclear which part of the organism would be best for tissue samples, so tail and ear clips were requested. Ear clips were thick with dense hair and skin, while the tail had shorter, less dense hair and more tissue. A matchstick‐sized tissue sample was cut and as much hair as possible was removed using a razor blade. Then the sample was prepared for DNA extraction by putting it in 2 mL of CTAB buffer with 100 μL of proteinase K and 6 μL of RNAse (from Maxwell RSC PureFood GMO and Authentication Kit, Promega). This mixture was added to a PowerWater bead beating tube (Qiagen) and vortexed horizontally for 5 min. at the maximum speed, followed by a 30 min. incubation at 60°C, another 5 min. vortex on the maximum speed, and finally a 2‐h incubation at 60°C. The resulting lysate was milky, so 1 mL was pulled out and subjected to centrifugation for 10 min. at 16,000 *g* and the supernatant was used downstream in a Maxwell RSC Instrument (Promega) with the Maxwell RSC PureFood GMO and Authentication Kit (Promega) following the recommended protocol. The DNA extract was quantified using a Quantus Fluorometer (Promega), which revealed that the tail sample consistently yielded twice the DNA concentration, so the tail DNA extracts were used for downstream analysis.

The tissue samples were diluted to 10 ng/μL and then further diluted with TE in a 10× serial dilution of 12 subsamples to calculate the limit of detection (LOD) and limit of quantification (LOQ). The concentration of the samples ranged from 1 ng/μL (standard 1) through 1E−11 ng/μL (standard 12). Standards 1–4 were run in triplicate, standards 5–8 were run with 6 replicates, and standards 9–12 were run with 4 replicates to maximize the replicate number in the areas around the predicted LOD/LOQ. Following Klymus et al. (2020), the Generic qPCR LOD/LOQ calculator (Merkes, 2019) was used to estimate LOD/LOQ. The TaqMan probe assay from Akamatsu et al. (2018) was used, (F: 5′‐CACTACAACAGCTTTTTCATCAATCAC‐3′, R: 5′‐TTCCTCGTCCAATGTGGAAGT‐3′, probe: 5′‐TGATTAATCCGTTATATACACGCT‐3′) labeled with FAM and ZEN/Iowa Black™ FQ quencher (Integrated DNA Technologies) to test the qPCR. The qPCR assay was run in 20 μL reactions, with 10 μL of PrimeTime Master Mix (IDT), 2 μL of nutria primer/probe mix (IDT), 2 μL of IPC primer/probe mix (TaqMan Exogenous Internal Positive Control Reagents, Applied Biosystems), 4 μL of DNA template, and 0.4 μL of IPC template DNA on a Step One Plus (Applied Biosystems) qPCR machine with 95°C for 20 s., followed by 95°C for 1 s. and 60°C for 20 s. over 50 cycles.

### 2.2 Field validation

**FIGURE 1:**
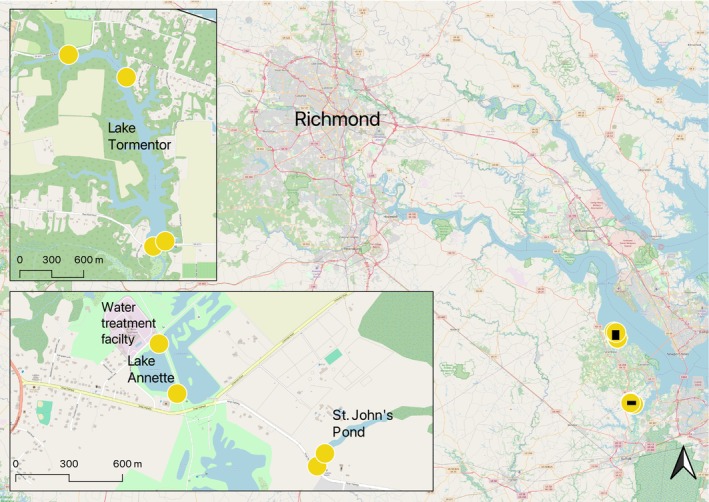
Map depicting the study area near Suffolk, VA in the United States. Inset maps show the location of sampling sites (circles) at St. John's Pond, Lake Annette, and Lake Tormentor.

**FIGURE 2:**
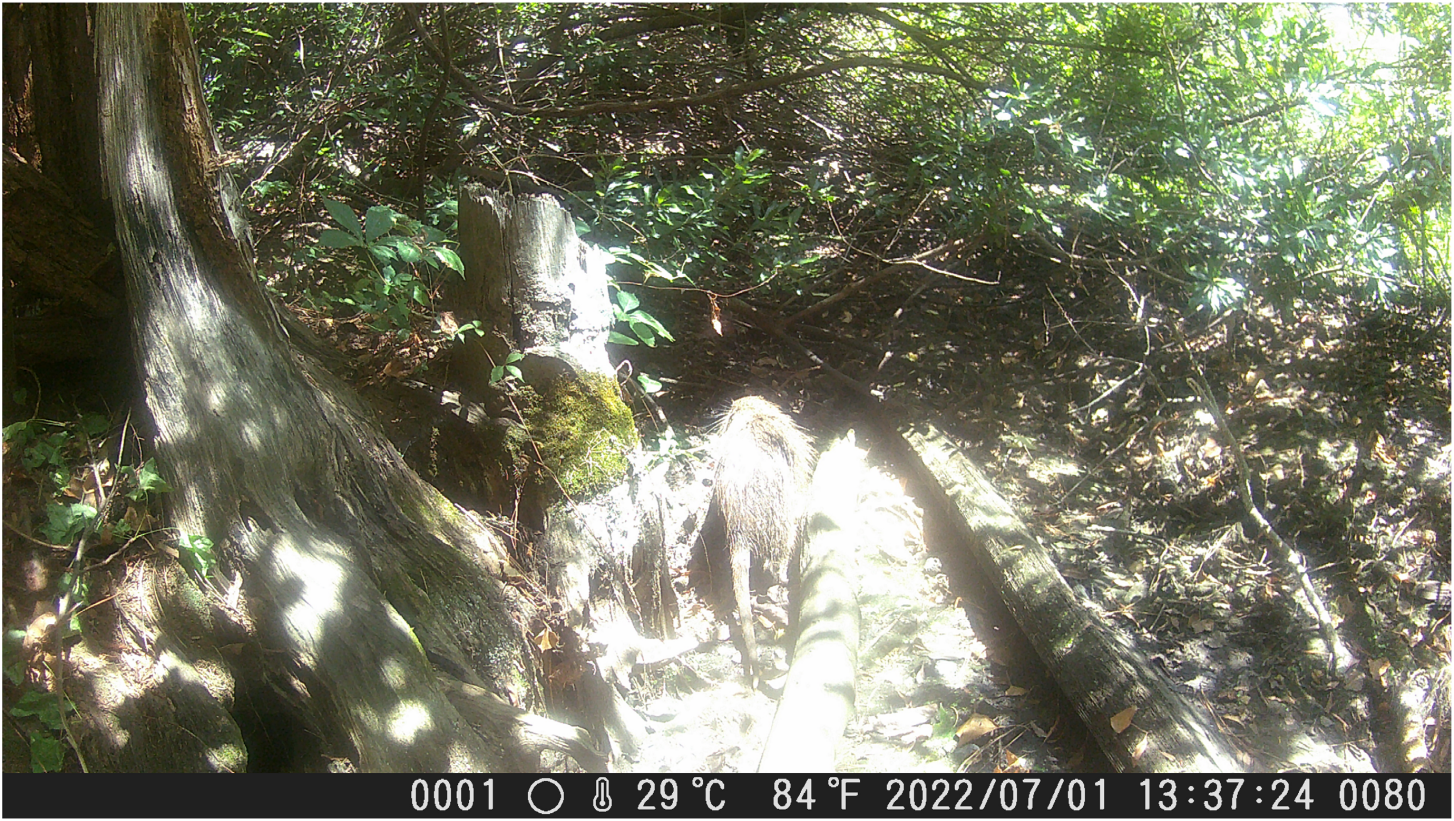
Nutria presence was confirmed by this photograph from the camera trap at St. John's Pond.

For field validation, several sites were sampled near Suffolk, VA (Figure 1). The study was designed to focus on a small pond (~7800 m^2^) with putative presence found adjacent to St. John's Episcopal church (hereafter called St. John's Pond). A camera trap was placed at the entrance to a putative nutria burrow for 3 weeks starting on June 29, 2022 to confirm nutria presence (Figure 2). For comparison, two sites were sampled for eDNA at a nearby pond with putative absence called Lake Annette (~36,000 m^2^) that provides inflow to a water treatment plant. At Lake Annette, sampling was conducted in December 2021 and January 2022. After confirming the absence of nutria, sampling was discontinued to conserve resources.

To assess seasonal variation in detection, St. John's Pond was sampled monthly from December 2021 to November 2022 and the water levels were categorized as “high,” “medium,” or “low” depending on how much standing water was in the pond. To assess diel variation, St. John's Pond was sampled in the morning and after sunset at the two sites monthly during June to August 2022. During the summer months (June to August), when field access and manpower were increased, monthly sampling was conducted at four sites around Lake Tormentor (~230,000 m^2^) where nutria harvest had occurred earlier in the year. This additional sampling aimed to explore whether the size of the water body influenced detection. Trends are reported in graphical form, but the small sample size precluded statistical analyses on diel or seasonal variation in detection.

Sampling consisted of collecting approximately 1 L of surface water from each site in Nalgene bottles that had been washed with a 50% bleach solution and rinsed thoroughly. Sampling took place using an improvised extension pole from the bank of the water source, with careful attention from the person sampling to avoid contact with the water. The water samples were filtered in the field through a Nalgene single use analytical filter funnel with a 0.45 μm cellulose nitrate filter (VWR) using a peristaltic pump until water stopped flowing through the filter. When the volume of filtrate was less than 500 mL, a second filter was used. One field negative control of purified water was filtered per day of sampling. When water levels in St. John's Pond dropped substantially during September to November 2022, only one water sample was captured, but this was supplemented with passive sampling consisting of placing a filter at the end of an improvised “fishing pole” and letting the filter sit in the water for approximately 1 h. To store filters, they were placed in a zip‐top bag holding silica desiccant and kept at room temperature until DNA extraction.

DNA extraction and qPCR set‐up took place in a laminar flow hood that was wiped down with LookOut DNA erase (Sigma‐Aldrich) and sterilized with UV light for 10 min. each session. Aerosol barrier tips were used throughout. The first step of DNA extraction consisted of putting the filter(s) (up to two from the same sampling location) in a bead beating tube, adding lysis buffer and vortexing the filters (using the same protocol as the above tissue extraction). Initially, PowerWater bead tubes were used with garnet beads (Qiagen), until they discontinued distribution of the garnet beads. After trying the replacement ceramic PowerWater beads with little success (they absorbed all the lysate), 2.8 mm ceramic beads in a 7 mL bead tube (Omni International) were used. All lysate was removed, centrifuged, and the DNA isolation protocol from the Maxwell RSC PureFood GMO and Authentication Kit (Promega) was followed.

For qPCR analysis, a seven‐step serial 10× dilution of the aforementioned tissue samples was used as a standard, ranging from 1E0 – 1E−6 ng/μL. All samples were run in triplicate, in two different plates for a total of six replicates following the qPCR protocol above with the addition of an internal positive control (IPC) using TaqMan Exogenous Internal Positive Control Reagents, (Applied Biosystems) to check for inhibition. Detection was considered positive when a minimum of one replicate fluoresced at a Ct value <38. On all plates, a no‐template control was run in addition to an IPC block to assess contamination. Inhibition was tested in each plate by comparing the average Ct value of the IPC from the no‐template controls to the average Ct value of the IPC from samples.

To confirm the absence of nutria from the water samples taken at St. John's Pond from August to November, an assay for the co‐occurring painted turtle (*Chrysemys picta*) was improved. Davy et al. (2015) created a Sybr Green eDNA assay for the painted turtle (CO1‐Cpi‐01‐F:GAAATTGACTCGTACCAATG, CO1‐Cpi‐01‐R:CACCCCTGCTAAGTGGAGAG), and tested this for sensitivity with other freshwater turtle species. To increase specificity, a probe labeled with FAM and ZEN/Iowa Black™ FQ quencher (Integrated DNA Technologies) was added: TCATCAGGAATTGAAGCAGGCGCA. The assay was tested on water filtered from two tanks at the Robins Nature Center at Maymont in Richmond, VA that housed painted turtles, and the extraction and qPCR procedures outlined above were followed with six technical replicates.

## 3 RESULTS

**FIGURE 3:**
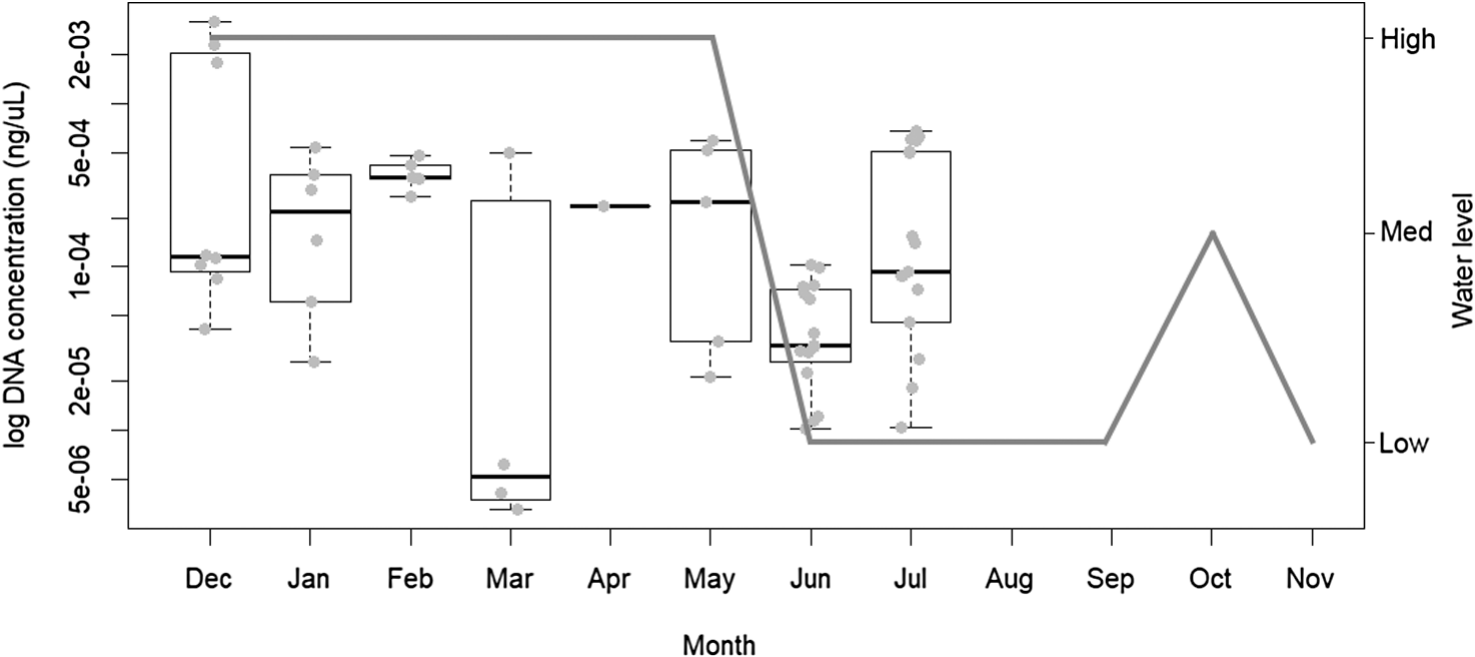
Concentration of nutria eDNA in ng/μL displayed on a log scale by month in St. John's Pond in Suffolk, VA. The gray line shows water levels by month. The bold horizontal lines of the boxplots represent medians, the edges represent the 25th and 75th percentile and the whiskers indicate the range of the data represented as 1.5 times the interquartile range.

DNA extracted from the nutria tissue samples was successfully amplified in the qPCR assay. The LOD and LOQ as estimated using the Generic LOD/LOQ calculator were the same, at a concentration of 1E−6 ng/μL. As there were several detections around the LOD and discarding these detections is not ideal, when the Ct value was <38, it was considered a positive detection. The field verification at St. John's Pond where nutria was present (confirmed via camera trapping) was successful, as detection was positive at multiple sampling events (Figure 3). Positive detections occurred monthly from December 2021 to July 2022, although not always at both sample sites within St. John's Pond. Nutria were not detected at all in St. John's Pond from August to November 2022. There were no nutria detections at Lake Annette, or at sites around Lake Tormentor (Table 1).

**TABLE 1:** Results of qPCR analysis of eDNA samples by species, site, month, amplification success, Ct mean and mean DNA quantity in ng/μL across samples.

Species	Site	Month	Amp. Success	Ct mean	Mean quantity (ng/μL)
*M. coypus*	St. John's Pond	Dec	8/16	34.9	9.04E−04
*M. coypus*	St. John's Pond	Jan	6/27	35.0	2.39E−04
*M. coypus*	St. John's Pond	Feb	5/12	34.4	3.75E−03
*M. coypus*	St. John's Pond	Mar	4/12	36.3	1.29E−04
*M. coypus*	St. John's Pond	Apr	1/12	37.2	2.37E−04
*M. coypus*	St. John's Pond	May	5/12	35.9	2.85E−04
*M. coypus*	St. John's Pond	Jun	15/24	34.9	4.71E−05
*M. coypus*	St. John's Pond	Jul	17/24	34.0	4.29E−03
*M. coypus*	St. John's Pond	Aug	0/24		
*M. coypus*	St. John's Pond	Sep	0/18		
*M. coypus*	St. John's Pond	Oct	0/24		
*M. coypus*	St. John's Pond	Nov	0/6		
*M. coypus*	Lake Annette	Dec to Jan	0/33		
*M. coypus*	Lake Tormentor	Jun to Aug	0/117		
*C. picta*	Tanks		10/12	30.8	2.70E+00
*C. picta*	St. John's Pond	Aug	6/6	32.8	3.10E+01
*C. picta*	St. John's Pond	Sept	9/9	36.9	1.13E−01
*C. picta*	St. John's Pond	Oct	3/6	35.8	2.90E−03
*C. picta*	St. John's Pond	Nov	3/3	34.6	7.33E−03

*Note*: Amplification success is the number of positive replicates (Ct < 38) out of the total number of replicates.

To confirm the absence of nutria, the water samples were re‐analyzed for the presence of painted turtle. First, the painted turtle assay was successful at amplifying the turtle eDNA from tanks at the Robins Nature Center at Maymont in Richmond, VA. The LOD and LOQ as estimated using the Generic LOD/LOQ calculator were the same, at a concentration 1E−7 ng/μL. The painted turtle assay also detected turtles at St. John's Pond monthly from August to November 2022, confirming that the eDNA sampling and analysis procedures worked.

**FIGURE 4:**
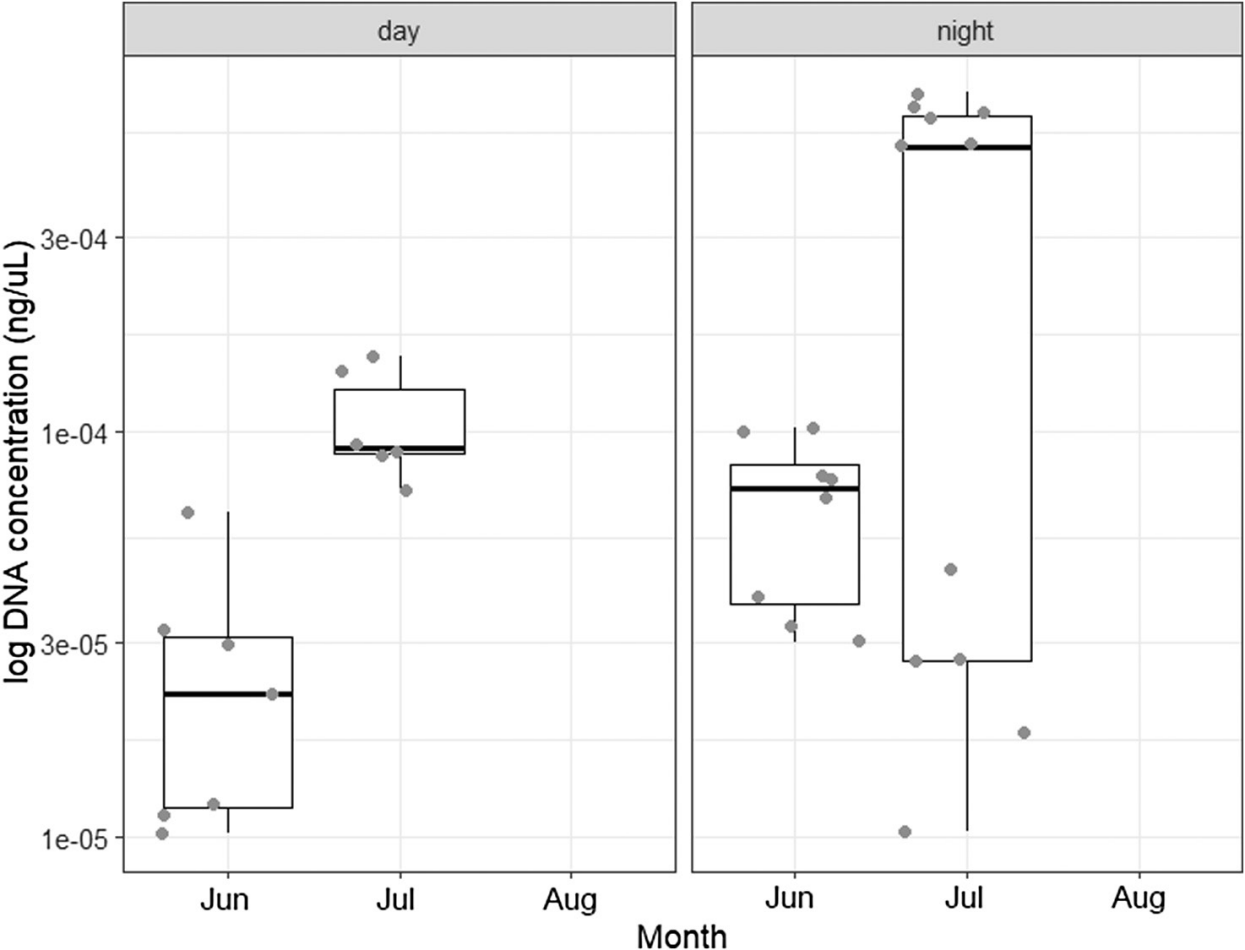
Concentration of nutria eDNA in ng/μL displayed on a log scale by month and time at St. John's Pond in Suffolk, VA, showing diel variation in DNA concentration, with the concentration slightly higher post‐sunset.

Passive sampling during Sept. 2022 did not amplify nutria DNA, which is congruent with the results from active sampling. When testing the painted turtle assay on the filters from passive sampling, there was positive detection in 5/6 replicates. Diel variation in detection of nutria was assessed June to August 2022, and found consistent detection at both times of day, with a trend for slightly higher eDNA concentrations from night sampling (Figure 4). All field negative controls and lab negative controls were negative, the efficiency of the qPCR assays averaged 93.6%, and no inhibition was detected.

## 4 DISCUSSION

This study provides support for the applicability of the eDNA assay described by Akamatsu et al. (2018) for field use in Virginia. Similar to Mangan et al. (2023), this study confirmed that the assay was able to detect nutria in the field, and that filtering water in the manner practiced was effective for DNA capture. The findings revealed a link between water levels and nutria detection, with higher water levels generally associated with higher concentrations of eDNA. At the lowest water level observed, the pond resembled little more than a puddle, rendering it inhospitable for sustaining significant pond life and prompting dispersal of the nutria. This finding emphasizes the influence of water levels on nutria behavior and highlights the potential for further research to characterize this relationship, which could ultimately yield valuable insights for defining nutria management strategies.

By utilizing the painted turtle assay as a follow‐up to negative detections of nutria, not only was the nutria's departure from St. John's Pond confirmed, but it was ascertained that they did not return throughout the duration of the study (August to November 2022). Additionally, the test with passive sampling, involving the placement of filters in the water for just 1 h, yielded positive detection of painted turtle eDNA, indicating promise for this sampling technique in future work.

Some eDNA concentrations for positive detections were smaller than the LOD. There is recognition that the LOD/LOQ as defined by MIQE (Bustin et al., 2009) can be a conservative measure that results in type II errors for studies exploring detection using eDNA (Agersnap et al., 2017; Klymus et al., 2020; Knudsen et al., 2019). As such, there may be inconsistent detection and imprecise quantification of eDNA at lower concentrations, but in this case, missing a detection is more problematic than poor quantification. Having the additional cutoff of a Ct value that defines the threshold at which detection can be qualitatively confirmed helps limit these type II errors (Klymus et al., 2020).

This study did not detect nutria at Lake Tormentor. This is convergent with the observation of a member of the public, who harvested 12 individuals in early 2022 and reported the area to be free of nutria since April, 2 months prior to sampling at this location. Further evaluation of the eDNA assay in diverse waterbodies of varying sizes and conditions, including brackish water environments, warrants exploration. Existing research has underscored the challenges associated with eDNA detection in brackish water due to inhibitory factors (Adrian‐Kalchhauser & Burkhardt‐Holm, 2016; Hunter et al., 2017), as well as in large waterbodies where eDNA dispersion patterns may differ substantially from those in confined ponds (Jane et al., 2015; Weldon et al., 2020). These considerations emphasize the need for continued investigation to refine the application of eDNA assays in different ecological contexts.

### 4.1 Implications for conservation

Collectively, these findings underscore the eDNA assay's sensitivity in discerning the absence of nutria and point to complete signal decay in the focal pond within a relatively short time frame (i.e., 1 month). This is a noteworthy finding, as it suggests that the eDNA assay could be a valuable monitoring tool post‐eradication. While the conventional means of using monitoring platforms and at‐site inspection by field personnel undoubtedly hold value and can offer a rapid response to invasion, using eDNA to monitor post‐eradication allows a few people to sample widely and detect the presence even when physical signs are lacking.

During the push to remove invasive species, financial resources can often support more people on the ground, but when eradication is complete, there are fewer resources to monitor for re‐invasion. This reduction in resources can be problematic, as nutria have become re‐established in areas such as California, USA and Italy after extensive eradication efforts (Bertolino et al., 2011; California Department of Fish and Game, 2023). This may be where an eDNA assay for nutria holds the most potential as a management tool. The process of eDNA sampling requires the involvement of only a few individuals to collect water samples, and sampling at multiple sites can be completed within a single day. The subsequent steps of DNA extraction and qPCR analysis can be carried out independent of sampling and many samples can be run at once, which improves efficiency. This process offers cost‐effective identification of local hotspots or re‐invasions that demand follow‐up eradication actions.

## AUTHOR CONTRIBUTIONS

**Stephanie S. Coster:** Conceptualization; data curation; formal analysis; funding acquisition; investigation; methodology; project administration; writing – original draft; writing – review and editing.

## FUNDING INFORMATION

This study was funded by a grant to SSC under award F18AP00243 from the U.S. Department of the Interior/U.S. Fish and Wildlife Service to Maryland Sea Grant, the administrative entity for the Mid‐Atlantic Panel on Aquatic Invasive Species. The statements, findings, conclusions, and recommendations are those of the author and do not necessarily reflect the views of Maryland Sea Grant, the U.S. Department of Interior, or the U.S. Fish and Wildlife Service.

## CONFLICT OF INTEREST STATEMENT

The author asserts no conflicts of interest in connection with this article.

## Data Availability

The data that support these findings can be found on Dryad at doi: 10.5061/dryad.ttdz08m56.

## References

[ece311416-bib-0001] Adrian‐Kalchhauser, I. , & Burkhardt‐Holm, P. (2016). An eDNA assay to monitor a globally invasive fish species from flowing freshwater. PLoS One, 11, e0147558. 10.1371/journal.pone.0147558 26814998 PMC4729461

[ece311416-bib-0002] Agersnap, S. , Larsen, W. B. , Knudsen, S. W. , Strand, D. , Thomsen, P. F. , Hesselsøe, M. , Mortensen, P. B. , Vrålstad, T. , & Møller, P. R. (2017). Monitoring of noble, signal and narrow‐clawed crayfish using environmental DNA from freshwater samples. PLoS One, 12, e0179261. 10.1371/journal.pone.0179261 28654642 PMC5487031

[ece311416-bib-0003] Akamatsu, Y. , Goto, M. , Emperor, Q. , Yamanaka, H. , Komuro, T. , & Kono, T. (2018). Understanding the invasion status and suitable habitat of nutria in the second‐class rivers in Yamaguchi prefecture using environmental DNA. Applied Ecological Engineering, 21, 1–8.

[ece311416-bib-0004] Anderson, D. P. , Pepper, M. A. , Travers, S. , Michaels, T. A. , Sullivan, K. , & Ramsey, D. S. L. (2022). Confirming the broadscale eradication success of nutria (*Myocastor coypus*) from the Delmarva Peninsula, USA. Biological Invasions, 24, 3509–3521. 10.1007/s10530-022-02855-x

[ece311416-bib-0005] Baker, S. J. , & Clarke, C. N. (1988). Cage trapping coypus (*Myocastor coypus*) on baited rafts. Journal of Applied Ecology, 25, 41–48. 10.2307/2403608

[ece311416-bib-0006] Bertolino, S. , Laura, G. M. , & Jacoby, C. (2011). *Mycastor coypus* Molina (coypu). In R. A. Francis (Ed.), A handbook of global freshwater invasive species (pp. 357–368). Routledge. 10.4324/9780203127230

[ece311416-bib-0007] Bustin, S. A. , Benes, V. , Garson, J. A. , Hellemans, J. , Huggett, J. , Kubista, M. , Mueller, R. , Nolan, T. , Pfaffl, M. W. , Shipley, G. L. , Vandesompele, J. , & Wittwer, C. T. (2009). The MIQE guidelines: Minimum information for publication of quantitative real‐time PCR experiments. Clinical Chemistry, 55, 611–622. 10.1373/clinchem.2008.112797 19246619

[ece311416-bib-0008] California Department of Fish and Game . (2023). California's invaders: Nutria . https://wildlife.ca.gov/Conservation/Invasives/Species/Nutria#:%7E:text%3DSubsequent%20introductions%20were%20successful%2C%20as,continent%20except%20Antarctica%20and%20Australia

[ece311416-bib-0009] Carter, J. , & Leonard, B. P. (2002). A review of the literature on the worldwide distribution, spread of, and efforts to eradicate the coypu (*Myocastor coypus*). Wildlife Society Bulletin, 30, 162–175.

[ece311416-bib-0010] Davy, C. M. , Kidd, A. G. , & Wilson, C. C. (2015). Development and validation of environmental DNA (eDNA) markers for detection of freshwater turtles. PLoS One, 10, e0130965. 10.1371/journal.pone.0130965 26200348 PMC4511736

[ece311416-bib-0011] Drake, J. M. (2005). Risk analysis for invasive species and emerging infectious diseases: Concepts and applications. American Midland Naturalist, 153, 4–19. 10.1674/0003-0031(2005)153[0004:RAFISA]2.0.CO;2

[ece311416-bib-0012] Elbrecht, V. , & Leese, F. (2017). Validation and development of COI metabarcoding primers for freshwater macroinvertebrate bioassessment. Frontiers in Environmental Science, 5, 11. 10.3389/fenvs.2017.00011

[ece311416-bib-0013] Ficetola, G. F. , Miaud, C. , Pompanon, F. , & Taberlet, P. (2008). Species detection using environmental DNA from water samples. Biology Letters, 4, 423–425. 10.1098/rsbl.2008.0118 18400683 PMC2610135

[ece311416-bib-0014] Goldberg, C. S. , Pilliod, D. S. , Arkle, R. S. , & Waits, L. P. (2011). Molecular detection of vertebrates in stream water: A demonstration using Rocky Mountain tailed frogs and Idaho giant salamanders. PLoS One, 6, e22746. 10.1371/journal.pone.0022746 21818382 PMC3144250

[ece311416-bib-0015] Holden, M. H. , Nyrop, J. P. , & Ellner, S. P. (2016). The economic benefit of time‐varying surveillance effort for invasive species management. Journal of Applied Ecology, 53, 712–721. 10.1111/1365-2664.12617

[ece311416-bib-0016] Hunter, M. E. , Dorazio, R. M. , Butterfield, J. S. S. , Meigs‐Friend, G. , Nico, L. G. , & Ferrante, J. A. (2017). Detection limits of quantitative and digital PCR assays and their influence in presence–absence surveys of environmental DNA. Molecular Ecology Resources, 17, 221–229. 10.1111/1755-0998.12619 27768244

[ece311416-bib-0017] Jane, S. F. , Wilcox, T. M. , McKelvey, K. S. , Young, M. K. , Schwartz, M. K. , Lowe, W. H. , Letcher, B. H. , & Whiteley, A. R. (2015). Distance, flow and PCR inhibition: eDNA dynamics in two headwater streams. Molecular Ecology Resources, 15, 216–227. 10.1111/1755-0998.12285 24890199

[ece311416-bib-0018] Jojola, S. , Witmer, G. , & Nolte, D. (2005). Nutria: an invasive rodent pest or valued resource? Wildlife Damage Management Conferences – Proceedings 120–126 . https://digitalcommons.unl.edu/icwdm_wdmconfproc/110

[ece311416-bib-0019] Kimberlin, J. (2021). They're rodents, of unusual size: War on invasive nutria heats up in Hampton roads, with collateral damage . The Virginian‐Pilot. https://www.pilotonline.com/life/wildlife‐nature/vp‐db‐nutria‐or‐muskrat‐053021‐20210530‐uvrux5337nawjptqzujh3fvoc4‐story.html

[ece311416-bib-0020] Klocke, B. , Becker, M. , Lewis, J. , Fleischer, R. C. , Muletz‐Wolz, C. R. , Rockwood, L. , Aguirre, A. A. , & Gratwicke, B. (2017). *Batrachochytrium salamandrivorans* not detected in U.S. survey of pet salamanders. Scientific Reports, 7, 13132. 10.1038/s41598-017-13500-2 29030586 PMC5640657

[ece311416-bib-0021] Klymus, K. E. , Merkes, C. M. , Allison, M. J. , Goldberg, C. S. , Helbing, C. C. , Hunter, M. E. , Jackson, C. A. , Lance, R. F. , Mangan, A. M. , Monroe, E. M. , Piaggio, A. J. , Stokdyk, J. P. , Wilson, C. C. , & Richter, C. A. (2020). Reporting the limits of detection and quantification for environmental DNA assays. Environmental DNA, 2, 271–282. 10.1002/edn3.29

[ece311416-bib-0022] Klymus, K. E. , Richter, C. A. , Chapman, D. C. , & Paukert, C. (2015). Quantification of eDNA shedding rates from invasive bighead carp *Hypophthalmichthys nobilis* and silver carp *Hypophthalmichthys molitrix* . Biological Conservation, Special Issue: Environmental DNA: A Powerful New Tool for Biological Conservation, 183, 77–84. 10.1016/j.biocon.2014.11.020

[ece311416-bib-0023] Knudsen, S. W. , Ebert, R. B. , Hesselsøe, M. , Kuntke, F. , Hassingboe, J. , Mortensen, P. B. , Thomsen, P. F. , Sigsgaard, E. E. , Hansen, B. K. , Nielsen, E. E. , & Møller, P. R. (2019). Species‐specific detection and quantification of environmental DNA from marine fishes in the Baltic Sea. Journal of Experimental Marine Biology and Ecology, 510, 31–45. 10.1016/j.jembe.2018.09.004

[ece311416-bib-0024] Langlois, V. S. , Allison, M. J. , Bergman, L. C. , To, T. A. , & Helbing, C. C. (2021). The need for robust qPCR‐based eDNA detection assays in environmental monitoring and species inventories. Environmental DNA, 3, 519–527. 10.1002/edn3.164

[ece311416-bib-0025] LeBlanc, D. J. (1994). Nutria. In S. E. Hygnstrom , R. M. Timm , & G. E. Larson (Eds.), The handbook: Prevention and control of wildlife damage (pp. B71–B80). USDA‐APHIS.

[ece311416-bib-0026] Mangan, A. M. , Kronenberger, J. A. , Plummer, I. H. , Wilcox, T. M. , & Piaggio, A. J. (2023). Validation of a nutria (*Myocastor coypus*) environmental DNA assay highlights considerations for sampling methodology. Environmental DNA, 5, 391–402.

[ece311416-bib-0027] Merkes, C. M. (2019). Generic qPCR limit of detection (LOD)/limit of quantification (LOQ) calculator . https://github.com/cmerkes/qPCR_LOD_Calc

[ece311416-bib-0028] Messina, R. (2021). Menace of the Marsh . Virginia Wildlife Magazine Nov/Dec 2021.

[ece311416-bib-0029] Pepper, M. , Herrmann, V. , Hines, J. , Nichols, J. , & Kendrot, S. (2017). Evaluation of nutria (*Myocastor coypus*) detection methods in Maryland, USA. Biological Invasions, 19, 831–841. 10.1007/s10530-016-1312-1

[ece311416-bib-0030] Sheffels, T. R. , & Sytsma, M. (2007). Report on nutria management and research in the Pacific northwest (technical report). Portland State University, Center for Lakes and Reservoirs. 10.15760/mem.32

[ece311416-bib-0031] Takahara, T. , Minamoto, T. , Yamanaka, H. , Doi, H. , & Kawabata, Z. (2012). Estimation of fish biomass using environmental DNA. PLoS One, 7, e35868. 10.1371/journal.pone.0035868 22563411 PMC3338542

[ece311416-bib-0032] Thalinger, B. , Deiner, K. , Harper, L. R. , Rees, H. C. , Blackman, R. C. , Sint, D. , Traugott, M. , Goldberg, C. S. , & Bruce, K. (2021). A validation scale to determine the readiness of environmental DNA assays for routine species monitoring. Environmental DNA, 3, 823–836.

[ece311416-bib-0033] Thomsen, P. F. , & Willerslev, E. (2015). Environmental DNA – An emerging tool in conservation for monitoring past and present biodiversity. Biological Conservation, Special Issue: Environmental DNA: A Powerful New Tool for Biological Conservation, 183, 4–18. 10.1016/j.biocon.2014.11.019

[ece311416-bib-0034] Turner, C. R. , Uy, K. L. , & Everhart, R. C. (2015). Fish environmental DNA is more concentrated in aquatic sediments than surface water. Biological Conservation, Special Issue: Environmental DNA: A Powerful New Tool for Biological Conservation, 183, 93–102. 10.1016/j.biocon.2014.11.017

[ece311416-bib-0035] Weldon, L. , O'Leary, C. , Steer, M. , Newton, L. , Macdonald, H. , & Sargeant, S. L. (2020). A comparison of European eel *Anguilla Anguilla* eDNA concentrations to fyke net catches in five Irish lakes. Environmental DNA, 2, 587–600. 10.1002/edn3.91

[ece311416-bib-0036] Williams, J. (2021). VA on alert for invasive nutria . Chesapeake Bay Magazine. https://chesapeakebaymagazine.com/va‐on‐alert‐for‐invasive‐nutria/

[ece311416-bib-0037] Zeale, M. R. K. , Butlin, R. K. , Barker, G. L. A. , Lees, D. C. , & Jones, G. (2011). Taxon‐specific PCR for DNA barcoding arthropod prey in bat faeces. Molecular Ecology Resources, 11, 236–244. 10.1111/j.1755-0998.2010.02920.x 21429129

